# Pan-Immune-Inflammation Value: A New Prognostic Index in Operative Breast Cancer

**DOI:** 10.3389/fonc.2022.830138

**Published:** 2022-04-13

**Authors:** Fei Lin, Li-Ping Zhang, Shuang-Yan Xie, Han-Ying Huang, Xiao-Yu Chen, Tong-Chao Jiang, Ling Guo, Huan-Xin Lin

**Affiliations:** ^1^ State Key Laboratory of Oncology in South China, Guangdong Key Laboratory of Nasopharyngeal Carcinoma Diagnosis and Therapy, Sun Yat-sen University Cancer Center, Collaborative Innovation Center for Cancer Medicine, Guangzhou, China; ^2^ Department of Radiotherapy, Sun Yat-sen University Cancer Center, Guangzhou, China; ^3^ Department of Nasopharyngeal Carcinoma, Sun Yat-sen University Cancer Center, Guangzhou, China; ^4^ Department of Oncology, Guangdong Province Hospital of Integrated of Traditional Chinese and Western Medicine, Foshan, China

**Keywords:** breast cancer, nomogram, PIV, index, prognosis

## Abstract

**Background:**

To build a predictive scoring model based on simple immune and inflammatory parameters to predict postoperative survival in patients with breast cancer.

**Methods:**

We used a brand-new immuno-inflammatory index—pan-immune-inflammation value (PIV)—to retrospectively evaluate the relationship between PIV and overall survival (OS), and based on the results of Cox regression analysis, we established a simple scoring prediction model based on several independent prognostic parameters. The predictive accuracy of the model was evaluated and independently validated.

**Results:**

A total of 1,312 patients were included for analysis. PIV was calculated as follows: neutrophil count (10^9^/L) × platelet count (10^9^/L) × monocyte count (10^9^/L)/lymphocyte count (10^9^/L). According to the best cutoff value of PIV, we divided the patients into two different subgroups, high PIV (PIV > 310.2) and low PIV (PIV ≤ 310.2), associated with significantly different survival outcomes (3-year OS, 80.26% vs. 86.29%, respectively; 5-year OS, 62.5% vs. 71.55%, respectively). Six independent prognostic factors were identified and used to build the scoring system, which performed well with a concordance index (C-index) of 0.759 (95% CI: 0.715–0.802); the calibration plot showed good calibration.

**Conclusions:**

We have established and verified a simple scoring system for predicting prognosis, which can predict the survival of patients with operable breast cancer. This system can help clinicians implement targeted and individualized treatment strategies.

## Introduction

Breast cancer is the most common malignant tumor in women (1, 2) and has the highest incidence among all malignancies affecting women according to the WHO (3). Although the overall survival (OS) rate of breast cancer has improved owing to advancements in diagnosis and treatment over the past decades (4), there is still a non-negligible fraction of patients with poor outcomes, and the latest study shows that some high-income countries report continuous substantial improvements exceeding 2% annual mortality reduction of breast cancer; however, many low- and middle-income countries have changeless or even increasing mortality rates (5, 6). Moreover, it is known that breast cancer patients with the same clinical stage and receiving the same treatment may have completely different outcomes. Because of such prognostic heterogeneity (7), carrying out individualized precision treatment is paramount to treatment success. Therefore, it is necessary to identify new and suitable alternative biomarkers for better prognostic stratification and prediction of treatment outcomes.

At present, many studies have proven that inflammatory factors such as IL-6 and TGF-β, inflammatory reactions, and the immune system are associated with the development and progression of two types of cancer attributable to chronic inflammatory disease: cholangiocarcinoma and colitis-associated colorectal cancer (8, 9). Research has shown that macrophages, essential components of the immune-inflammatory response, are implicated in inflammatory mechanisms and can therefore facilitate tumorigenesis in colorectal cancer (10). In addition, studies have demonstrated that hematologic parameters such as lymphocyte level are promising biomarkers of the body’s immune and inflammation status (11). In recent years, research on immune-inflammatory biomarkers (IIBs), compared with traditional tumor-related biomarkers, that can affect the prognosis of breast cancer has shown significant progress. Several easy-to-obtain and blood-based IIBs have been proven as potential independent prognostic factors in breast cancer, such as neutrophil-to-lymphocyte ratio (NLR), platelet-to-lymphocyte ratio (PLR), and systemic immune-inflammation index (SII) (12–16).

Because of the complex interactions between the tumor and host immune-inflammatory responses (17), the abovementioned indicators based on simple calculations inevitably limit the prediction power of the prognosis. The pan-immune-inflammation value (PIV), a new comprehensive biomarker involving the neutrophil, platelet, monocyte, and lymphocyte counts, has been proven to be a strong predictor of survival outcomes with better performance than other well-known IIBs in patients with metastatic colorectal cancer (18). However, the prognostic value of PIV is rarely reported in breast cancer. Therefore, this study aimed to clarify the prognostic value of PIV in breast cancer.

## Methods

### Patients

In all, 1,312 patients were included in this retrospective study who underwent surgery at the Sun Yat-sen University Cancer Center (SYSUCC; Guangzhou, China) between December 2010 and October 2012. The inclusion criteria were as follows: 1) pathologically confirmed breast cancer and 2) receipt of mastectomy or lumpectomy. The exclusion criteria were as follows: 1) relapse and *de novo* breast cancer; 2) complicated with another primary tumor; 3) ductal carcinoma *in situ* (DCIS); 4) male breast cancer; 5) receipt of any antitumor treatment before surgery; 6) concurrent hematological, autoimmune, or acute/chronic inflammatory disease; 7) incomplete laboratory data resulting in the non-calculation of the PIV indicator; and 8) follow-up loss. This study was approved by the Research Ethics Committee of SYSUCC. All patients’ data were confidential.

### Data Collection and Definitions

The list of patients who visited our hospital was obtained from the follow-up department. Then the patient’s laboratory data were checked through the case system and recorded in Excel in detail. Laboratory data were collected 1 week before surgery (at first diagnosis, before any treatment), and clinicopathological data were collected from the patients’ medical records. The calculation formula of each indicator was as follows: SII = platelet count (10^9^/L) × neutrophil count/lymphocyte count (10^9^/L); NLR = neutrophil count (10^9^/L)/lymphocyte count (10^9^/L); PLR = platelet count (10^9^/L)/lymphocyte count (10^9^/L) (19); and PIV = neutrophil count (10^9^/L) × platelet count (10^9^/L) × monocyte count (10^9^/L)/lymphocyte count (10^9^/L) (20). According to the calculation formula mentioned above, the PIV and other indicators were calculated in Excel, and the sorted data were analyzed for further statistical analysis using R. Patients were staged according to the eighth edition American Joint Committee on Cancer—Tumor, Node, and Metastases (AJCC-TNM) staging system (21). The expression of estrogen receptor (ER) and progesterone receptor (PR) were scored using the St. Gallen criteria (22). Human epidermal growth factor receptor-2 (HER-2) status was assessed according to the American Society of Clinical Oncology–College of American Pathologists guidelines (23, 24) by using immunohistochemistry or fluorescence *in situ* hybridization (FISH) test. HER-2-negative status was defined as immunohistochemistry showing HER-2+/++, or the FISH test results are negative, or the FISH test was not performed; HER-2-positive status was defined as immunohistochemical staining = 3+ or FISH positive/chromogenic *in situ* hybridization positive.

### Follow-Up

Follow-up was performed telephonically or through a regular outpatient surveillance system to record the condition of patients or the cause and date of death if the patient had already died. In this study, the endpoint was OS—defined as the time between the date of diagnosis and death due to any reason. The date of the last follow-up was considered the study endpoint for all surviving patients. The date of the last follow-up was considered for patients who did not reach the study endpoint.

### Statistical Analysis

Continuous variables were presented as the median and interquartile range (IQR). Categorical variables were presented as frequency and percentage. A chi-square test and the Mann–Whitney U test were used to analyze the association between PIV groups and other clinicopathological characteristics. In this research, a two-tailed *p*-value <0.05 was considered to indicate statistical significance. Maximally selected rank statistics were used to determine the optimal cutoff of continuous variables. Survival curves were plotted using the Kaplan–Meier method, and significance was determined by the log-rank test. All factors with a *p*-value <0.05 detected in univariate analyses were entered into the multivariate model to identify independent prognostic factors. Before multivariate analyses, the proportional hazards assumption test was performed using the Schoenfeld residuals. The variables with *p*-value <0.05 in the multivariate analyses were finally selected to build a prognostic model, which was presented as a nomogram. Concordance index (C-index) was used to evaluate the predictive accuracy of the nomogram. The calibration curves were used to predict the ability of the calibration between the predicted and actual survival. To avoid overfitting, 1,000 bootstrap samples and 10-fold cross-validation were also applied. All analyses were performed with R software (http://www.R-project.org; version 4.0.2) and SPSS 25.0 (IBM Corporation, Armonk, NY, USA).

## Results

### The Optimal Cutoff Value of Pan-Immune-Inflammation Value

The optimal cutoff value for PIV was 310.2 in the whole cohort by using maximally selected rank statistics (**Supplementary Figure 1**).

### Patient Characteristics and Relationship Between Pan-Immune-Inflammation Value and Clinicopathological Factors

A total of 1,312 breast cancer patients were enrolled in this study. The relationship between clinicopathological characteristics and PIV of the whole cohort is presented in **Table 1**. Briefly, the median age of the patients was 48 years (IQR, 41–57). From the perspective of the clinical stage, 317 (24.2%), 679 (51.7%), and 316 (24.1%) patients were diagnosed with stage I, II, and III cancer, respectively. Overall, 1,109 (84.5%) patients had invasive ductal carcinoma, and 203 (15.5%) had other pathological types. The median body mass index (BMI) of the patients was 23 (IQR, 20.8–25.2). The median follow-up time was 78.4 months (IQR, 53.1–88). The median PIV of the patients was 135.2 (IQR, 87.6–213.7). Further, 387 (29.5%) patients were HER-2 positive, and 925 (70.5%) were negative. The median values of the pretreatment platelet count, neutrophil count, monocyte count, and lymphocyte count were 225 × 10^9^/L, 3.65 × 10^9^/L, 0.32 × 10^9^/L, and 1.9 × 10^9^/L, respectively.

**Table 1:** The relationship between PIV and clinicopathological characteristics in the whole cohort.

Characteristic	Total (N = 1,312)	High-PIV group (N = 152)	Low-PIV group (N = 1,160)	*p*
**Age (years), median (IQR)**	48 (41–57)	46 (40–55)	48 (41–57)	0.127
**Tumor stage**				0.292
T1	467 (35.6)	52 (34.2)	415 (35.8)	
T2	719 (54.8)	79 (52.0)	640 (55.2)	
T3	65 (5.0)	10 (6.6)	55 (4.7)	
T4	61 (4.6)	11 (7.2)	50 (4.3)	
**Node stage**				0.178
N0	687 (52.4)	69 (45.4)	618 (53.3)	
N1	345 (26.3)	42 (27.6)	303 (26.1)	
N2	163 (12.4)	26 (17.1)	137 (11.8)	
N3	117 (8.9)	15 (9.9)	102 (8.8)	
**Clinical stage**				–
I	317 (24.2)	30 (19.7)	287 (24.7)	
II	679 (51.7)	73 (48.0)	606 (52.3)	
III	316 (24.1)	49 (32.3)	267 (23.0)	
**BMI kg/m^2^, median (IQR)**	23 (20.8–25.2)	23.4 (21.0–25.7)	22.9 (20.8–25.1)	0.158
**Histological type**				0.902
Invasive ductal carcinoma	1,109 (84.5)	129 (84.9)	980 (84.5)	
Others	203 (15.5)	23 (15.1)	180 (15.5)	
**ER status**				0.020*
Positive	942 (71.8)	97 (63.8)	845 (72.8)	
Negative	370 (28.2)	55 (36.2)	315 (27.2)	
**PR status**				0.413
Positive	842 (64.2)	93 (61.2)	749 (64.6)	
Negative	470 (35.8)	59 (38.8)	411 (35.4)	
**HER-2 status**				0.975
Positive	387 (29.5)	45 (29.6)	342 (29.5)	
Negative	925 (70.5)	107 (70.4)	818 (70.5)	
**Ki-67**				0.349
>14%	575 (43.8)	72 (47.4)	503 (43.4)	
≤14%	737 (56.2)	80 (52.6)	657 (56.6)	
**Adjuvant chemotherapy**				0.285
Yes	1,066 (81.3)	111 (73.0)	955 (82.3)	
No	246 (18.7)	41 (27.0)	205 (17.7)	
**Radiotherapy**				0.761
Yes	350 (26.7)	43 (28.3)	307 (26.5)	
No	962 (73.3)	109 (71.7)	853 (73.5)	
**Endocrine therapy**				0.818
Yes	680 (51.8)	79 (52.0)	601 (51.8)	
No	632 (48.2)	73 (48.0)	559 (48.2)	
**Target therapy**				0.485
Yes	95 (7.2)	15 (9.9)	80 (6.9)	
No	1,217 (92.8)	137 (90.1)	1,080 (93.1)	
**PLT (10^9^/L), median (IQR)**	225 (190.0–265.0)	272 (236.9–310.5)	220.5 (186.0–255.2)	–
**NE (10^9^/L), median (IQR)**	3.7 (2.9–4.6)	5.3 (4.4–7.0)	3.5 (2.8–4.3)	–
**MONO (10^9^/L), median (IQR)**	0.3 (0.2–0.4)	0.5 (0.4–0.6)	0.3 (0.2–0.4)	–
**LY (10^9^/L), median (IQR)**	1.9 (1.6–2.3)	1.63 (1.4–2.2)	1.91 (1.6–2.3)	–

PIV low group (PIV ≤ 310.2) and PIV high group (PIV > 310.2).

PIV, pan-immune-inflammation value; IQR, interquartile range; BMI, body mass index; ER, estrogen receptor; PR, progesterone receptor; HER-2, human epidermal growth factor receptor-2; PLT, platelet count; NE, neutrophil count; MONO, monocyte count; LY, lymphocyte count. *p < 0.05.

The analysis of the relationship between PIV and various clinicopathological factors showed that PIV was significantly associated with ER status (*p* = 0.02).

The whole cohort was randomly divided into a training set and a validation set (ratio: 7:3) (**Table 2**). With respect to the PIV group, 819 (89.0%) and 341 (87.0%) patients were assigned to the low-PIV group in the training set and validation set, respectively.

**Table 2:** The baseline characteristics between the training and validation datasets.

Characteristics	Training set (N = 920)	Validation set (N = 392)
**Age (years), median (IQR)**	48 (42–57)	47 (44–55)
**Tumor stage**		
T1	330 (35.9%)	137 (35.0%)
T2	508 (55.2%)	211 (53.8%)
T3	39 (4.2%)	26 (6.6%)
T4	43 (4.7%)	18 (4.6%)
**Node stage**		
N0	470 (51.0%)	217 (55.4%)
N1	254 (27.6%)	91 (23.2%)
N2	123 (13.4%)	40 (10.2%)
N3	73 (8.0%)	44 (11.2%)
**Clinical stage**		
I	222 (24.2%)	95 (24.2%)
II	476 (51.7%)	203 (51.8%)
III	222 (24.1%)	94 (24.0%)
**Histological type**		
Invasive ductal carcinoma	781 (84.9%)	328 (83.7%)
Others	139 (15.1%)	64 (16.3%)
**ER status**		
Positive	661 (71.8%)	281 (71.7%)
Negative	259 (28.2%)	111 (28.3%)
**PR status**		
Positive	591 (64.2%)	251 (64.0%)
Negative	329 (35.8%)	141 (36.0%)
**HER-2 status**		
Positive	202 (22.0%)	82 (20.9%)
Negative	718 (78.0%)	310 (79.1%)
**Ki-67**		
>14%	403 (43.8%)	172 (43.9%)
≤14%	517 (56.2%)	220 (56.1%)
PIV		
>310.2	101 (11.0%)	51 (13.0%)
≤31.02	819 (89.0%)	341 (87.0%)

IQR, interquartile range; ER, estrogen receptor; PR, progesterone receptor; HER-2, human epidermal growth factor receptor-2.

### Survival Analysis of Pan-Immune-Inflammation Value Groups

**Figure 1:**
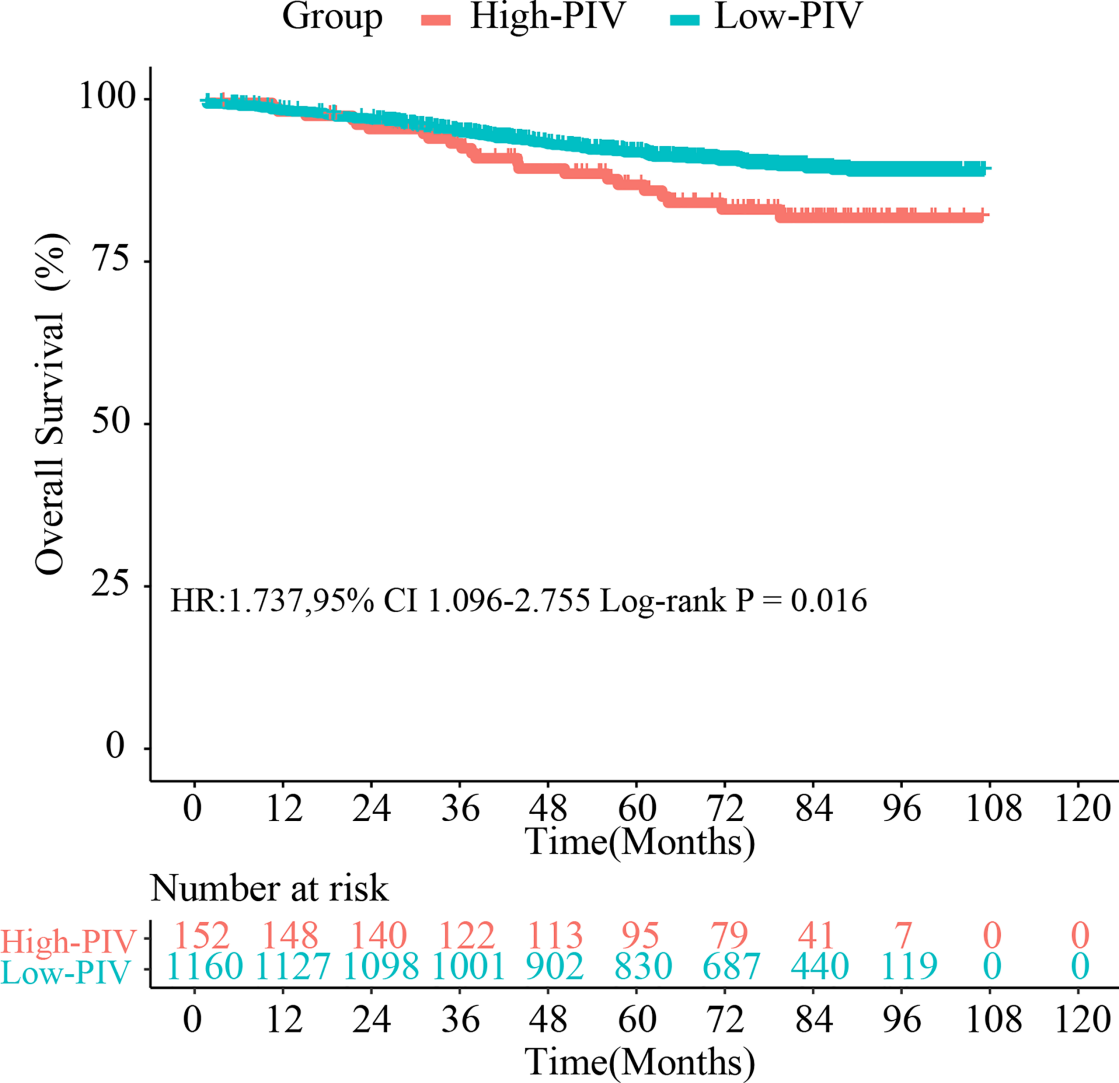
Kaplan–Meier survival curves with breast cancer after surgery between the high-PIV group and low-PIV group in the whole cohort. PIV, pan-immune-inflammation value.

According to the optimal cutoff value of PIV, the whole cohort was divided into two groups: the low-PIV group (PIV ≤ 310.2) and the high-PIV group (PIV > 310.2). **Figure 1** shows the significant survival differences between the two groups. The 3-year OS rates in the low-PIV group and the high-PIV group were 86.29% and 80.26%, respectively; the 5-year OS rates in the low-PIV group and the high-PIV group were 71.55% and 62.50%, respectively (hazard ratio (HR): 1.737, 95% CI: 1.096–2.755, log-rank test, *p* = 0.016).

Moreover, we performed univariate and multivariate Cox regression analyses for OS. Indicators that related to breast cancer clinically and common IIBs such as NLR, PLR, and SII were selected in the univariate analysis. The results have shown that T stage, N stage, histopathological type, ER status, PR status, HER-2 status, Ki-67, NLR, and PIV were potential factors associated with OS (**Table 3**). The global *p*-value was 0.231 of the PH-test, which means that the constructed multi-regression analysis model is successful. The abovementioned indicators were further analyzed in the multivariable Cox regression analysis. In the final model, we observed that T stage, N stage, histopathological type, PR status, Ki-67, and PIV were significant independent prognostic factors of breast cancer (**Table 3**), which is graphically presented as **Supplementary Figure 2**.

**Table 3:** Univariate and multivariate analyses of overall survival.

Characteristic	Univariate analysisHazard ratio (95% CI)	*p*	Multivariate analysisHazard ratio (95% CI)	*p*
**Age (years)**	1.153 (0.811–1.640)	0.427	–	–
**T stage^#^**	2.415 (1.547–3.771)	<0.001^*^	1.633 (1.027–2.596)	0.038^*^
**N stage^#^**	5.572 (3.823–8.121)	<0.001^*^	4.719 (3.195–6.971)	<0.001^*^
**Histopathological Type**	2.674 (1.306–5.473)	0.007^*^	2.668 (1.302–5.468)	0.007^*^
**ER status**	0.572 (0.399–0.822)	0.002^*^	0.902 (0.521–1.563)	0.713
**PR status**	0.568 (0.399–0.808)	0.002^*^	0.695 (0.483–0.998)	0.049^*^
**HER-2 status**	1.691 (1.181–2.421)	0.004^*^	1.231 (0.845–1.793)	0.279
**Ki-67**	2.197 (1.526–3.162)	<0.001^*^	1.713 (1.175–2.497)	0.005^*^
**NLR group**	1.440 (1.0122.051)	0.043^*^	1.598 (0.574–2.365)	0.064
**PLR group**	1.488 (0.981–2.232)	0.062	–	–
**SII group**	1.356 (0.903–2.037)	0.142	–	–
**PIV group**	1.737 (1.096–2.755)	0.016^*^	1.720 (1.083–2.730)	0.021^*^

A Cox proportional hazards model was used to conduct multivariate analyses. All variables were transformed into categorical variables. HRs of variables were calculated as follows: Age (>48 vs. ≤48 years); T stage (T1 vs. T234); N stage (N012 vs. N3); histological Type (invasive ductal carcinoma vs. others); ER (negative vs. positive); PR (negative vs. positive); HER-2 (negative vs. positive); Ki-67 (≤14% vs. >14%); NLR group (≤1.99 vs. >1.99); PLR group (≤160.25 vs. >160.25); SII group (≤642.23 vs. >642.23); PIV group (≤310.20 vs. >310.20).

HR, hazard ratio; ER, estrogen receptor; PR, progesterone receptor; HER-2, human epidermal growth factor receptor-2; NLR, neutrophil-to-lymphocyte ratio; PLR, platelet-to-lymphocyte ratio; SII, systemic immune-inflammation index; PIV, pan-immune-inflammation value.

^#^According to the eighth edition of the Union for International Cancer Control/American Joint Committee on Cancer (UICC/AJCC) staging system.

^*^p < 0.05.

In the training set and validation set, we conducted survival analyses and univariate and multivariate Cox regression analyses (**Tables S1**, **S2**). The Kaplan–Meier survival curves of the training cohort and validation cohort are presented in **Supplementary Figure 3** (training cohort, HR: 1.831, 95% CI: 1.077–3.111, log-rank test, *p* = 0.021; validation cohort, HR: 1.687, 95% CI: 1.156–3.068, log-rank test, *p* = 0.024). The results of survival analysis were consistent with those of the whole set (all log-rank *p* < 0.05). The results of univariate analysis and multivariate Cox regression analyses were in line with the whole set as well.

### Prognostic Analysis and Building the Model

**Figure 2:**
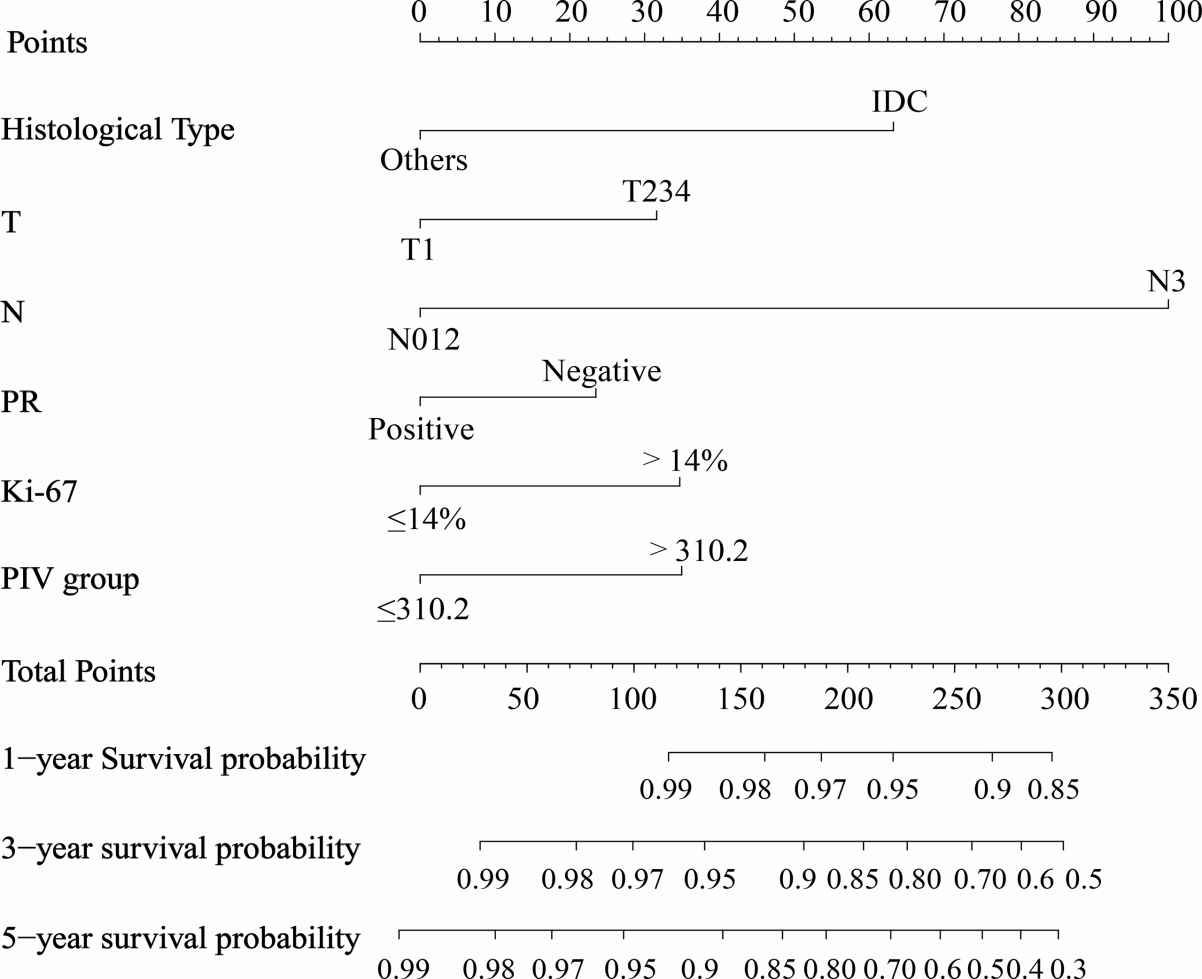
Nomogram to predict 1-, 3-, and 5-year overall survival generated using the whole cohort. IDC, invasive ductal carcinoma.

**Figure 3:**
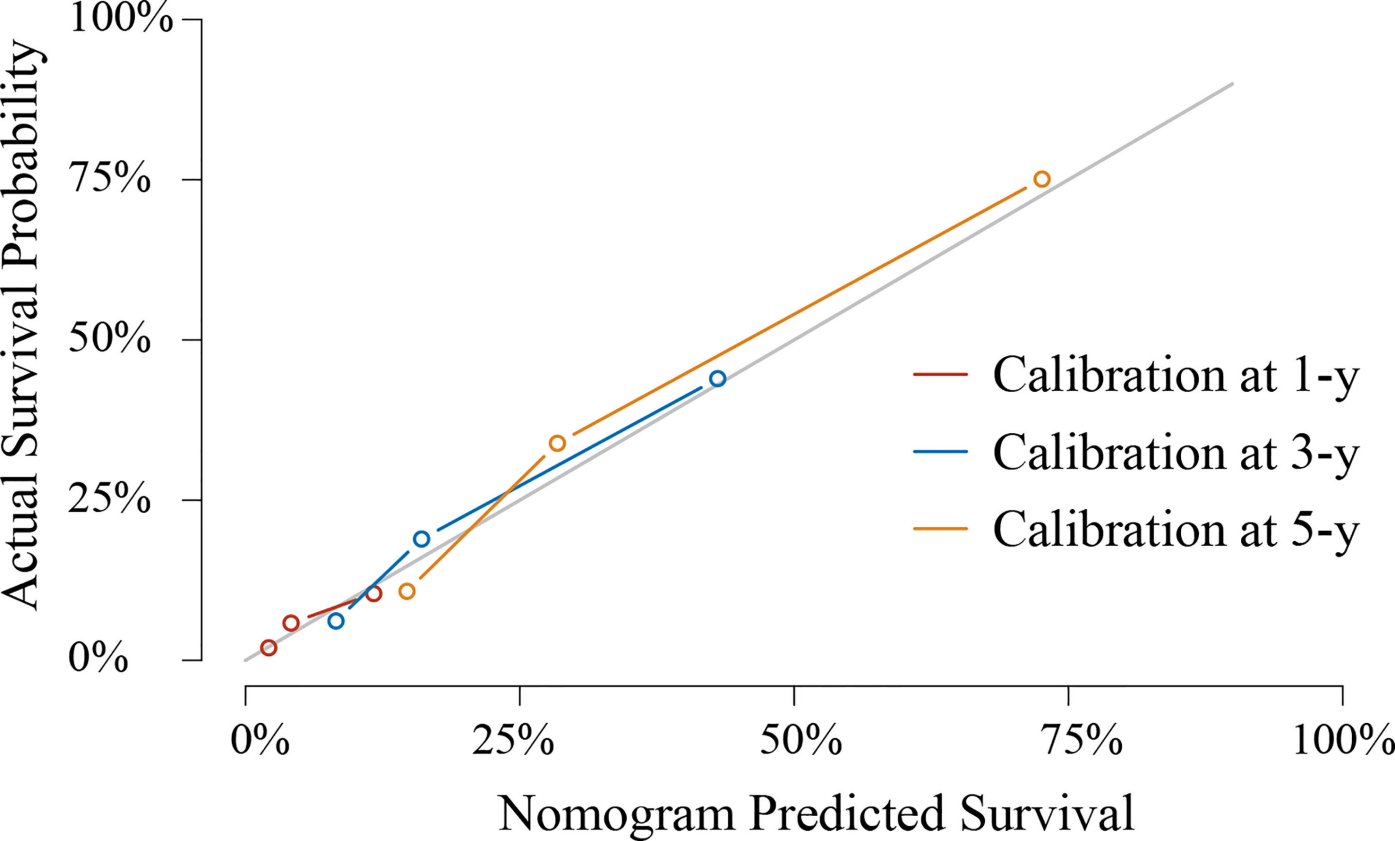
Calibration curves to predict 1-, 3-, and 5-year overall survival in the whole cohort.

Based on the abovementioned independent factors, a prognostic model for the prediction of the 1-, 3-, and 5-year OS was built and graphically presented as a nomogram (**Figure 2**). The prognostic model showed a good discriminating ability for OS prediction, with a C-index of 0.759 (95% CI: 0.715–0.802). The calibration curves of 1-, 3-, and 5-year OS illustrated good calibration between the predicted and actual survival probabilities in the whole cohort (**Figure 3**).

### Subgroup Analysis of Common Clinical Variables

**Figure 4:**
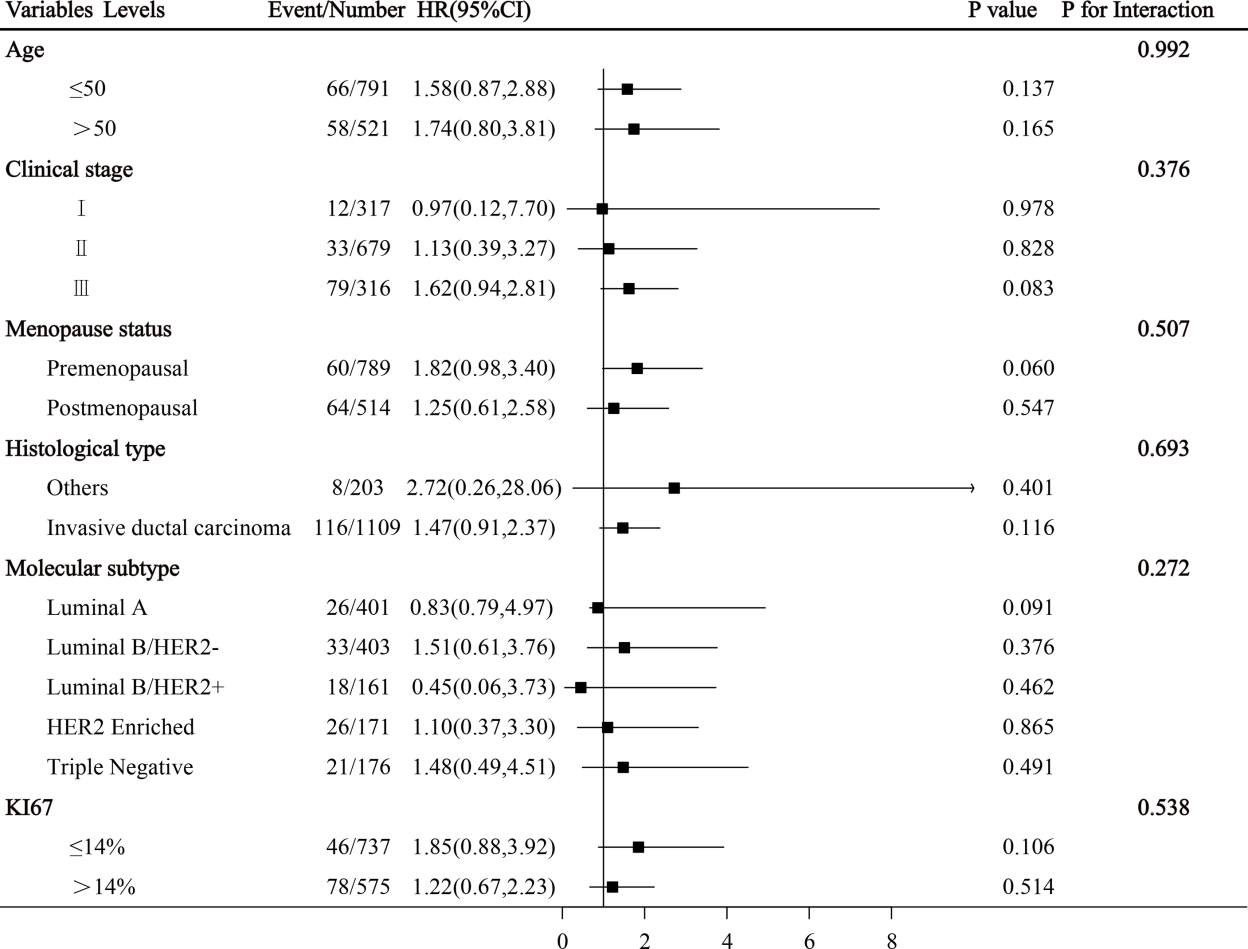
Subgroup analysis of breast cancer-related clinical variables were illustrated in a forest plot.

Subgroup analysis shows that there was no interaction between PIV and clinicopathological characteristics in the whole cohort (all *p* > 0.05, **Figure 4**).

## Discussion

The concept of tumor immunoediting includes the following three phases: elimination, equilibrium, and escape (25). The mechanism of immune escape is very complicated, which involves tumor-associated antigens, tumor gene mutation, several types of immune cells, an inflammatory microenvironment, and a tumor microenvironment (TME) (26). The TME includes not only the tumor cells but also immune and inflammatory cells (27, 28). One study showed that tumor cells interact with platelets both inside the TME and in the bloodstream or ascitic fluid (29). Another study reported that neutrophils promote tumor cell growth and progression by secreting cytokines and chemokines so as to offer a proper microenvironment for tumor cells (30). Tumor-associated macrophages (TAMs) are derived from circulating monocytes and play a crucial role in the formation of TME by promoting tumor progression and metastasis (31). The characteristics of the TME are hypoxia, chronic inflammation, and immunosuppression, which make a more complex network mechanism to regulate the relationship between systemic inflammation, local immune response, cancer progression, and patient survival (32–34).

In the current study, we used real-world data to assess the prognostic value of the PIV in operable breast cancer. The results showed that PIV, a new immune-inflammation score, was an independent predictor for breast cancer. Another study on PIV in metastatic colorectal cancer arrived at a similar conclusion as ours (18). Patients with low PIV have a better prognosis than those with high. What is more, we compared the effectiveness of PIV and the traditional TNM staging system in predicting prognosis by using time-dependent receiver operating characteristic (ROC) analysis; it revealed that PIV had higher accuracy in predicting OS than the traditional TNM staging system (**Supplementary Figure 4**), further highlighting the clinical application value of PIV.

In routine clinical work, for breast cancer, clinicians often determine the treatment according to molecular subtype, gene expression features, and clinical stage. In our study, subgroup analysis showed that there was no interaction between PIV and clinicopathological characteristics, which proves that the PIV has predictive consistency for each subgroup statistically. Of course, its real clinical application value needs to be confirmed by larger-scale data and prospective studies in the future.

The results of correlation analysis showed a relationship between PIV and ER status; the exact reason why PIV and ER status are significantly associated remains unclear. One of the possible reasons is selection bias. Although this association is statistically significant, it remains to be seen in future studies whether there is a true clinical relevance.

Several studies also have shown that patients with high levels of NLR, PLR, and SII have a poor prognosis in operable breast cancer (14, 35–37). The conclusion is as follows: NLR, PLR, and SII were independent prognostic factors in these studies. These findings were different from ours. As to NLR in our study, patients with low NLR showed a better prognosis than those with high NLR (**Supplementary Figure 5**), which is consistent with other studies (14) (38, 39). However, further multivariate Cox regression analysis showed that NLR is not an independent prognostic factor for breast cancer patients, while PIV is. The reason for this difference remains unclear. Perhaps, the small sample size of patients in this study did not allow us draw a conclusion between NLR and independent prognostic factors. As to PLR and SII, the univariate analysis, showed that neither PLR nor SII was a potential factor associated with OS for breast cancer in our study. The relationship between NLR, PLR, SII, and breast cancer prognosis is complex: many reports concluded that NLR (14, 40, 41), PLR (37, 42, 43), and SII (16, 44, 45) were independent prognostic factors for breast cancer, but there are also many studies that do not support the conclusion mentioned above (46–49). This may be related to the selection of the population and the included variables. In our current research, 1,312 patients were included in this retrospective study who underwent surgery at the Sun Yat-sen University Cancer Center between December 2010 and October 2012, and we included a new variable, PIV, a novel indicator of combined immuno-inflammation nutrition; time-dependent ROC curves show that PIV has better prognostic value than NLR, PLR, and SII (**Supplementary Figure 6**).

As PIV is a relatively novel biomarker, few studies on PIV have been reported thus far. A previous study (50) showed that a low PIV value predicts better chemotherapy response and survival in breast cancer patients treated with neoadjuvant chemotherapy. Another previous study (20) showed that PIV is a new and potent predictor of OS in HER-2-positive advanced BC patients treated with first-line trastuzumab–pertuzumab-containing biochemotherapy.

Our study has some limitations. First, there were inevitable flaws due to the nature of the retrospective observational design (51). Second, there were a relatively limited number of patients enrolled in this study. Third, patients included in this research were from a single cancer center. Therefore, potential selection bias could have led to data not being representative of the true distribution of PIV values in the whole cohort.

What is more, there was a very important point that the methods of obtaining the optimal cutoff value of PIV varied among studies. One study (52) used the median value of this parameter in the clinical cohort, while others (53–55) used the ROC curve to obtain the optimal value. In this study, we classified the candidate continuous index according to the cutoff point determined by the maximally selected rank statistics using the “maxstat” package of R software (56), a widely recognized and applied method in many studies (57–59). Thus, the cutoff value of PIV varies among studies, which limited the clinical use of this biomarker. The cutoff value determined in this study needs more research for further verification.

In addition, it must be mentioned that though we have established the model by randomly dichotomizing into the training and testing groups (at 7:3) in our study, we established the model by using the training cohort and validated the model by using the testing cohort. Also, we have made multifaceted efforts to validate our results. We tried to use different cohorts from public databases to validate the findings outlined in this study. Public databases such as the Surveillance, Epidemiology, and End Results (SEER) database and The Cancer Genome Atlas (TCGA) database were tried for validation, but none of them provided laboratory data (platelet counts, etc.). To the best of our knowledge, there are no available published public databases containing routine preoperative laboratory data. Also, we have been seeking data help from colleagues at Sun Yat-sen Memorial Hospital and Guangdong Provincial People’s Hospital in China, as well as from the organization Korean Breast Cancer Society in Korea. There are still some difficulties; regrettably, we have not obtained enough external validation data to confirm our findings so far. But we are actively seeking cooperation from other centers to verify the results. This is a limitation that should be considered.

The main strength of this study is that we believe we have supplemented the current knowledge of supporting evidence that PIV is independently related to survival outcomes in patients with breast cancer. We hope that future studies can further validate and confirm the application of the PIV indicator to other cancers as well.

## Conclusion

The PIV appears to be an independent predictor of OS in patients with operable breast cancer. The proposed nomogram could be a useful tool for individualized assessment of prognosis.

## Data Availability Statement

The original contributions presented in the study are included in the article/**Supplementary Material**. Further inquiries can be directed to the corresponding authors.

## Ethics Statement

In the present study, all the procedures involving human participants were performed according to the ethical standards of the institutional research committee or the national research committee, or both, and with the 1964 Declaration of Helsinki and its later amendments or comparable ethical standards. The Clinical Research Ethics Committee of SYSUCC approved this study (number: GZR2021-117). All participants in the study provided informed consent.

## Author Contributions

FL, H-XL, and LG contributed to literature search, study design, data analysis, writing, and critical revision. S-YX, H-YH, X-YC, and T-CJ: data acquisition, methodology, and data analysis. H-YH and L-PZ: visualization, investigation, and software. All authors: writing—reviewing, supervision, and editing.

## Funding

H-XL is supported by the National Natural Science Foundation of China (Grant No. 81773103) and the Natural Science Foundation of Guangdong Province (2017A030313617). LG is supported by the National Natural Science Foundation of China (Grant Nos. 81772877 and 81572848).

## Conflict of Interest

The authors declare that the research was conducted in the absence of any commercial or financial relationships that could be construed as a potential conflict of interest.

## Publisher’s Note

All claims expressed in this article are solely those of the authors and do not necessarily represent those of their affiliated organizations, or those of the publisher, the editors and the reviewers. Any product that may be evaluated in this article, or claim that may be made by its manufacturer, is not guaranteed or endorsed by the publisher.

## Supplementary Material

Supplementary Figure 1 The optimal cut-off of PIV for overall survival in the whole cohort by using maximally selected rank statistics.

Supplementary Figure 2 The forest plot of the results of multivariable regression analysis.

Supplementary Figure 3 Kaplan-Meier survival curves with breast cancer after surgery between the high-PIV group and low-PIV group **(A)** in the training set and **(B)** in the validation set.

Supplementary Figure 4 Time-dependent ROC curve compared with PIV and the TNM staging system.

Supplementary Figure 5 Kaplan-Meier survival curves of breast cancer patients after surgery between the high-NLR group and low-NLR group in the whole cohort.

Supplementary Figure 6 Time-dependent ROC curve compared with PIV and **(A)** NLR, **(B)** PLR, **(C)** SII.

## References

[1] Siegel RL Miller KD Jemal A. Cancer Statistics, 2020. CA Cancer J Clin (2020) 70(1):7–30. doi: 10.3322/caac.21590 31912902

[2] Loibl S Poortmans P Morrow M Denkert C Curigliano G. Breast Cancer. Lancet (2021) 397(10286):1750–69. doi: 10.1016/S0140-6736(20)32381-3 33812473

[3] Sung H Ferlay J Siegel RL Laversanne M Soerjomataram I Jemal A. Global Cancer Statistics 2020: GLOBOCAN Estimates of Incidence and Mortality Worldwide for 36 Cancers in 185 Countries. CA Cancer J Clin (2021) 71(3):209–49. doi: 10.3322/caac.21660 33538338

[4] Sun YS Zhao Z Yang ZN Xu F Lu HJ Zhu ZY. Risk Factors and Preventions of Breast Cancer. Int J Biol Sci (2017) 13(11):1387–97. doi: 10.7150/ijbs.21635 PMC5715522 29209143

[5] Trapani D Ginsburg O Fadelu T Lin NU Hassett M Ilbawi AM. Global Challenges and Policy Solutions in Breast Cancer Control. Cancer Treat Rev (2022) 104:102339. doi: 10.1016/j.ctrv.2022.102339 35074727

[6] Siegel RL Miller KD Fuchs HE Jemal A. Cancer Statistics, 2022. CA Cancer J Clin (2022) 72(1):7–33. doi: 10.3322/caac.21708 35020204

[7] Yeo SK Guan JL. Breast Cancer: Multiple Subtypes Within a Tumor? Trends Cancer (2017) 3(11):753–60. doi: 10.1016/j.trecan.2017.09.001 PMC5802368 29120751

[8] Landskron G de la Fuente M Thuwajit P Thuwajit C Hermoso MA. Chronic Inflammation and Cytokines in the Tumor Microenvironment. J Immunol Res (2014) 2014:149185. doi: 10.1155/2014/149185 24901008 PMC4036716

[9] Greten FR Grivennikov SI. Inflammation and Cancer: Triggers, Mechanisms, and Consequences. Immunity (2019) 51(1):27–41. doi: 10.1016/j.immuni.2019.06.025 31315034 PMC6831096

[10] Scheurlen KM Billeter AT O'Brien SJ Galandiuk S. Metabolic Dysfunction and Early-Onset Colorectal Cancer - How Macrophages Build the Bridge. Cancer Med (2020) 9(18):6679–93. doi: 10.1002/cam4.3315 PMC7520341 33624450

[11] Ma X Jia C Fu D Chu M Ding X Wu X. Analysis of Hematological Traits in Polled Yak by Genome-Wide Association Studies Using Individual SNPs and Haplotypes. Genes (Basel) (2019) 10(6):463. doi: 10.3390/genes10060463 PMC6627507 31212963

[12] Guo W Lu X Liu Q Zhang T Li P Qiao W. Prognostic Value of Neutrophil-to-Lymphocyte Ratio and Platelet-to-Lymphocyte Ratio for Breast Cancer Patients: An Updated Meta-Analysis of 17079 Individuals. Cancer Med (2019) 8(9):4135–48. doi: 10.1002/cam4.2281 PMC6675722 31197958

[13] Graziano V Grassadonia A Iezzi L Vici P Pizzuti L Barba M. Combination of Peripheral Neutrophil-to-Lymphocyte Ratio and Platelet-to-Lymphocyte Ratio Is Predictive of Pathological Complete Response After Neoadjuvant Chemotherapy in Breast Cancer Patients. Breast (2019) 44:33–8. doi: 10.1016/j.breast.2018.12.014 30611095

[14] Ethier JL Desautels D Templeton A Shah PS Amir E. Prognostic Role of Neutrophil-to-Lymphocyte Ratio in Breast Cancer: A Systematic Review and Meta-Analysis. Breast Cancer Res (2017) 19(1):2. doi: 10.1186/s13058-016-0794-1 28057046 PMC5217326

[15] Zhang Y Sun Y Zhang Q. Prognostic Value of the Systemic Immune-Inflammation Index in Patients With Breast Cancer: A Meta-Analysis. Cancer Cell Int (2020) 20:224. doi: 10.1186/s12935-020-01308-6 32528232 PMC7282128

[16] Hua X Long ZQ Zhang YL Wen W Guo L Xia W. Prognostic Value of Preoperative Systemic Immune-Inflammation Index in Breast Cancer: A Propensity Score-Matching Study. Front Oncol (2020) 10:580. doi: 10.3389/fonc.2020.00580 32373539 PMC7186330

[17] Nikolaou K Sarris M Talianidis I. Molecular Pathways: The Complex Roles of Inflammation Pathways in the Development and Treatment of Liver Cancer. Clin Cancer Res (2013) 19(11):2810–6. doi: 10.1158/1078-0432.CCR-12-1961 23549874

[18] Fuca G Guarini V Antoniotti C Morano F Moretto R Corallo S. The Pan-Immune-Inflammation Value Is a New Prognostic Biomarker in Metastatic Colorectal Cancer: Results From a Pooled-Analysis of the Valentino and TRIBE First-Line Trials. Br J Cancer (2020) 123(3):403–9. doi: 10.1038/s41416-020-0894-7 PMC7403416 32424148

[19] Peng RR Liang ZG Chen KH Li L Qu S Zhu XD. Nomogram Based on Lactate Dehydrogenase-To-Albumin Ratio (LAR) and Platelet-To-Lymphocyte Ratio (PLR) for Predicting Survival in Nasopharyngeal Carcinoma. J Inflamm Res (2021) 14:4019–33. doi: 10.2147/JIR.S322475 PMC8385134 34447260

[20] Ligorio F Fuca G Zattarin E Lobefaro R Zambelli L Leporati R. The Pan-Immune-Inflammation-Value Predicts the Survival of Patients With Human Epidermal Growth Factor Receptor 2 (HER2)-Positive Advanced Breast Cancer Treated With First-Line Taxane-Trastuzumab-Pertuzumab. Cancers (Basel) (2021) 13(8):1964. doi: 10.3390/cancers13081964 33921727 PMC8073809

[21] Zhang J Zhao B Jin F. The Assessment of 8th Edition AJCC Prognostic Staging System and a Simplified Staging System for Breast Cancer: The Analytic Results From the SEER Database. Breast J (2019) 25(5):838–47. doi: 10.1111/tbj.13347 31192530

[22] Carlson RW Hudis CA Pritchard KI National Comprehensive Cancer Network Breast Cancer Clinical Practice Guidelines in O American Society of Clinical Oncology Technology Assessment on the Use of Aromatase I St Gallen International Expert Consensus on the Primary Therapy of Early Breast C. Adjuvant Endocrine Therapy in Hormone Receptor-Positive Postmenopausal Breast Cancer: Evolution of NCCN, ASCO, and St Gallen Recommendations. J Natl Compr Canc Netw (2006) 4(10):971–9. doi: 10.6004/jnccn.2006.0082 17112447

[23] Wolff AC Hammond ME Hicks DG Dowsett M McShane LM Allison KH. Recommendations for Human Epidermal Growth Factor Receptor 2 Testing in Breast Cancer: American Society of Clinical Oncology/College of American Pathologists Clinical Practice Guideline Update. J Clin Oncol (2013) 31(31):3997–4013. doi: 10.1200/JCO.2013.50.9984 24101045

[24] Wolff AC Hammond MEH Allison KH Harvey BE Mangu PB Bartlett JMS. Human Epidermal Growth Factor Receptor 2 Testing in Breast Cancer: American Society of Clinical Oncology/College of American Pathologists Clinical Practice Guideline Focused Update. J Clin Oncol (2018) 36(20):2105–22. doi: 10.1200/JCO.2018.77.8738 29846122

[25] Dunn GP Old LJ Schreiber RD. The Three Es of Cancer Immunoediting. Annu Rev Immunol (2004) 22:329–60. doi: 10.1146/annurev.immunol.22.012703.104803 15032581

[26] Anichini A Perotti VE Sgambelluri F Mortarini R. Immune Escape Mechanisms in Non Small Cell Lung Cancer. Cancers (Basel) (2020) 12(12):3605. doi: 10.3390/cancers12123605 PMC7761620 33276569

[27] Hanahan D Weinberg RA. Hallmarks of Cancer: The Next Generation. Cell (2011) 144(5):646–74. doi: 10.1016/j.cell.2011.02.013 21376230

[28] Grivennikov SI Greten FR Karin M. Immunity, Inflammation, and Cancer. Cell (2010) 140(6):883–99. doi: 10.1016/j.cell.2010.01.025 PMC2866629 20303878

[29] Haemmerle M Taylor ML Gutschner T Pradeep S Cho MS Sheng J. Platelets Reduce Anoikis and Promote Metastasis by Activating YAP1 Signaling. Nat Commun (2017) 8(1):310. doi: 10.1038/s41467-017-00411-z 28827520 PMC5566477

[30] Gregory AD Houghton AM. Tumor-Associated Neutrophils: New Targets for Cancer Therapy. Cancer Res (2011) 71(7):2411–6. doi: 10.1158/0008-5472.CAN-10-2583 21427354

[31] Zhou SL Zhou ZJ Hu ZQ Huang XW Wang Z Chen EB. Tumor-Associated Neutrophils Recruit Macrophages and T-Regulatory Cells to Promote Progression of Hepatocellular Carcinoma and Resistance to Sorafenib. Gastroenterology (2016) 150(7):1646–58 e17. doi: 10.1053/j.gastro.2016.02.040 26924089

[32] Yu PF Huang Y Han YY Lin LY Sun WH Rabson AB. TNFalpha-Activated Mesenchymal Stromal Cells Promote Breast Cancer Metastasis by Recruiting CXCR2(+) Neutrophils. Oncogene (2017) 36(4):482–90. doi: 10.1038/onc.2016.217 PMC5290040 27375023

[33] Szkandera J Gerger A Liegl-Atzwanger B Absenger G Stotz M Samonigg H. Validation of the Prognostic Relevance of Plasma C-Reactive Protein Levels in Soft-Tissue Sarcoma Patients. Br J Cancer (2013) 109(9):2316–22. doi: 10.1038/bjc.2013.595 PMC3817333 24084772

[34] Nguyen AV Wu YY Lin EY. STAT3 and Sphingosine-1-Phosphate in Inflammation-Associated Colorectal Cancer. World J Gastroenterol (2014) 20(30):10279–87. doi: 10.3748/wjg.v20.i30.10279 PMC4130835 25132744

[35] Hu B Yang XR Xu Y Sun YF Sun C Guo W. Systemic Immune-Inflammation Index Predicts Prognosis of Patients After Curative Resection for Hepatocellular Carcinoma. Clin Cancer Res (2014) 20(23):6212–22. doi: 10.1158/1078-0432.CCR-14-0442 25271081

[36] Mouchli M Reddy S Gerrard M Boardman L Rubio M. Usefulness of Neutrophil-to-Lymphocyte Ratio (NLR) as a Prognostic Predictor After Treatment of Hepatocellular Carcinoma. Review Article. Ann Hepatol (2021) 22:100249. doi: 10.1016/j.aohep.2020.08.067 32896610

[37] Al Jarroudi O El Bairi K Abda N Zaimi A Jaouani L Chibani H. Neutrophil-To-Lymphocyte and Platelet-to-Lymphocyte Ratios as Predictors of Outcomes in Inflammatory Breast Cancer. Biomark Med (2021) 15(14):1289–98. doi: 10.2217/bmm-2020-0717 34486882

[38] Cupp MA Cariolou M Tzoulaki I Aune D Evangelou E Berlanga-Taylor AJ. Neutrophil to Lymphocyte Ratio and Cancer Prognosis: An Umbrella Review of Systematic Reviews and Meta-Analyses of Observational Studies. BMC Med (2020) 18(1):360. doi: 10.1186/s12916-020-01817-1 33213430 PMC7678319

[39] Moldoveanu D Pravongviengkham V Best G Martinez C Hijal T Meguerditchian AN. Dynamic Neutrophil-To-Lymphocyte Ratio: A Novel Prognosis Measure for Triple-Negative Breast Cancer. Ann Surg Oncol (2020) 27(10):4028–34. doi: 10.1245/s10434-020-08302-2 32314154

[40] Moon G Noh H Cho IJ Lee JI Han A. Prediction of Late Recurrence in Patients With Breast Cancer: Elevated Neutrophil to Lymphocyte Ratio (NLR) at 5 Years After Diagnosis and Late Recurrence. Breast Cancer (2020) 27(1):54–61. doi: 10.1007/s12282-019-00994-z 31280452

[41] Orlandini LF Pimentel FF Andrade JM Reis F Mattos-Arruda L Tiezzi DG. Obesity and High Neutrophil-to-Lymphocyte Ratio Are Prognostic Factors in Non-Metastatic Breast Cancer Patients. Braz J Med Biol Res (2021) 54(10):e11409. doi: 10.1590/1414-431X2021e11409 34406210 PMC8373197

[42] Cho U Park HS Im SY Yoo CY Jung JH Suh YJ. Prognostic Value of Systemic Inflammatory Markers and Development of a Nomogram in Breast Cancer. PloS One (2018) 13(7):e0200936. doi: 10.1371/journal.pone.0200936 30048474 PMC6062056

[43] Krenn-Pilko S Langsenlehner U Thurner EM Stojakovic T Pichler M Gerger A. The Elevated Preoperative Platelet-to-Lymphocyte Ratio Predicts Poor Prognosis in Breast Cancer Patients. Br J Cancer (2014) 110(10):2524–30. doi: 10.1038/bjc.2014.163 PMC4021515 24675383

[44] Zhu M Chen L Kong X Wang X Li X Fang Y. The Systemic Immune-Inflammation Index Is an Independent Predictor of Survival in Breast Cancer Patients. Cancer Manag Res (2022) 14:775–820. doi: 10.2147/CMAR.S346406 35241935 PMC8887616

[45] Jiang L Fang J Ding J. High Systemic Immune-Inflammation Index Predicts Poor Survival in Patients With Human Epidermal Growth Factor Receptor-2 Positive Breast Cancer Receiving Adjuvant Trastuzumab. Cancer Manag Res (2020) 12:475–84. doi: 10.2147/CMAR.S231444 PMC6982528 32021460

[46] Liu C Huang Z Wang Q Sun B Ding L Meng X. Usefulness of Neutrophil-to-Lymphocyte Ratio and Platelet-to-Lymphocyte Ratio in Hormone-Receptor-Negative Breast Cancer. Onco Targets Ther (2016) 9:4653–60. doi: 10.2147/OTT.S106017 PMC4973777 27536129

[47] Jiang C Lu Y Zhang S Huang Y. Systemic Immune-Inflammation Index Is Superior to Neutrophil to Lymphocyte Ratio in Prognostic Assessment of Breast Cancer Patients Undergoing Neoadjuvant Chemotherapy. BioMed Res Int (2020) 2020:7961568. doi: 10.1155/2020/7961568 33381583 PMC7762645

[48] Hu Y Wang S Ding N Li N Huang J Xiao Z. Platelet/Lymphocyte Ratio Is Superior to Neutrophil/Lymphocyte Ratio as a Predictor of Chemotherapy Response and Disease-Free Survival in Luminal B-Like (HER2(-)) Breast Cancer. Clin Breast Cancer (2020) 20(4):e403–9. doi: 10.1016/j.clbc.2020.01.008 32201163

[49] Takeuchi H Kawanaka H Fukuyama S Kubo N Hiroshige S Yano T. Comparison of the Prognostic Values of Preoperative Inflammation-Based Parameters in Patients With Breast Cancer. PloS One (2017) 12(5):e0177137. doi: 10.1371/journal.pone.0177137 28489884 PMC5425200

[50] Sahin AB Cubukcu E Ocak B Deligonul A Oyucu Orhan S Tolunay S. Low Pan-Immune-Inflammation-Value Predicts Better Chemotherapy Response and Survival in Breast Cancer Patients Treated With Neoadjuvant Chemotherapy. Sci Rep (2021) 11(1):14662. doi: 10.1038/s41598-021-94184-7 34282214 PMC8289916

[51] Kim HJ Park HS Go YJ Koh WU Kim H Song JG. Effect of Anesthetic Technique on the Occurrence of Acute Kidney Injury After Total Knee Arthroplasty. J Clin Med (2019) 8(6):778. doi: 10.3390/jcm8060778 PMC6616515 31159309

[52] Chen X Hong X Chen G Xue J Huang J Wang F. The Pan-Immune-Inflammation Value Predicts the Survival of Patients With Anaplastic Lymphoma Kinase-Positive Non-Small Cell Lung Cancer Treated With First-Line ALK Inhibitor. Transl Oncol (2022) 17:101338. doi: 10.1016/j.tranon.2021.101338 34999541 PMC8749135

[53] Demir H Demirci A Eren SK Beypinar I Davarci SE Baykara M. A New Prognostic Index in Young Breast Cancer Patients. J Coll Phys Surg Pak (2022) 32(1):86–91. doi: 10.29271/jcpsp.2022.01.86 34983154

[54] Gambichler T Said S Abu Rached N Scheel CH Susok L Stranzenbach R. Pan-Immune-Inflammation Value Independently Predicts Disease Recurrence in Patients With Merkel Cell Carcinoma. J Cancer Res Clin Oncol (2022). doi: 10.1007/s00432-022-03929-y PMC9508022 35098389

[55] Sato S Shimizu T Ishizuka M Suda K Shibuya N Hachiya H. The Preoperative Pan-Immune-Inflammation Value Is a Novel Prognostic Predictor for With Stage I-III Colorectal Cancer Patients Undergoing Surgery. Surg Today (2022). doi: 10.1007/s00595-021-02448-6 35015151

[56] Hothorn T Zeileis A. Generalized Maximally Selected Statistics. Biometrics (2008) 64(4):1263–9. doi: 10.1111/j.1541-0420.2008.00995.x 18325074

[57] Zhang B Xu H Zhang H Liu Q Ye Y Hao J. Prognostic Value of N-Terminal Pro-B-Type Natriuretic Peptide in Elderly Patients With Valvular Heart Disease. J Am Coll Cardiol (2020) 75(14):1659–72. doi: 10.1016/j.jacc.2020.02.031 32273031

[58] Jawitz NG Raman V Jawitz OK Shimpi RA Wood RK Hartwig MG. Utilization Trends and Volume-Outcomes Relationship of Endoscopic Resection for Early Stage Esophageal Cancer. Ann Surg (2021). doi: 10.1097/SLA.0000000000004834 PMC8966412 33914478

[59] Corti F Lonardi S Intini R Salati M Fenocchio E Belli C. The Pan-Immune-Inflammation Value in Microsatellite Instability-High Metastatic Colorectal Cancer Patients Treated With Immune Checkpoint Inhibitors. Eur J Cancer (2021) 150:155–67. doi: 10.1016/j.ejca.2021.03.043 33901794

