# Supplementary figures and images for: Pan-Immune-Inflammation Value: A New Prognostic Index in Operative Breast Cancer
Source: Front Oncol. 2022 Apr 13;12:830138. doi: 10.3389/fonc.2022.830138 (PMC9043599; doi:10.3389/fonc.2022.830138)

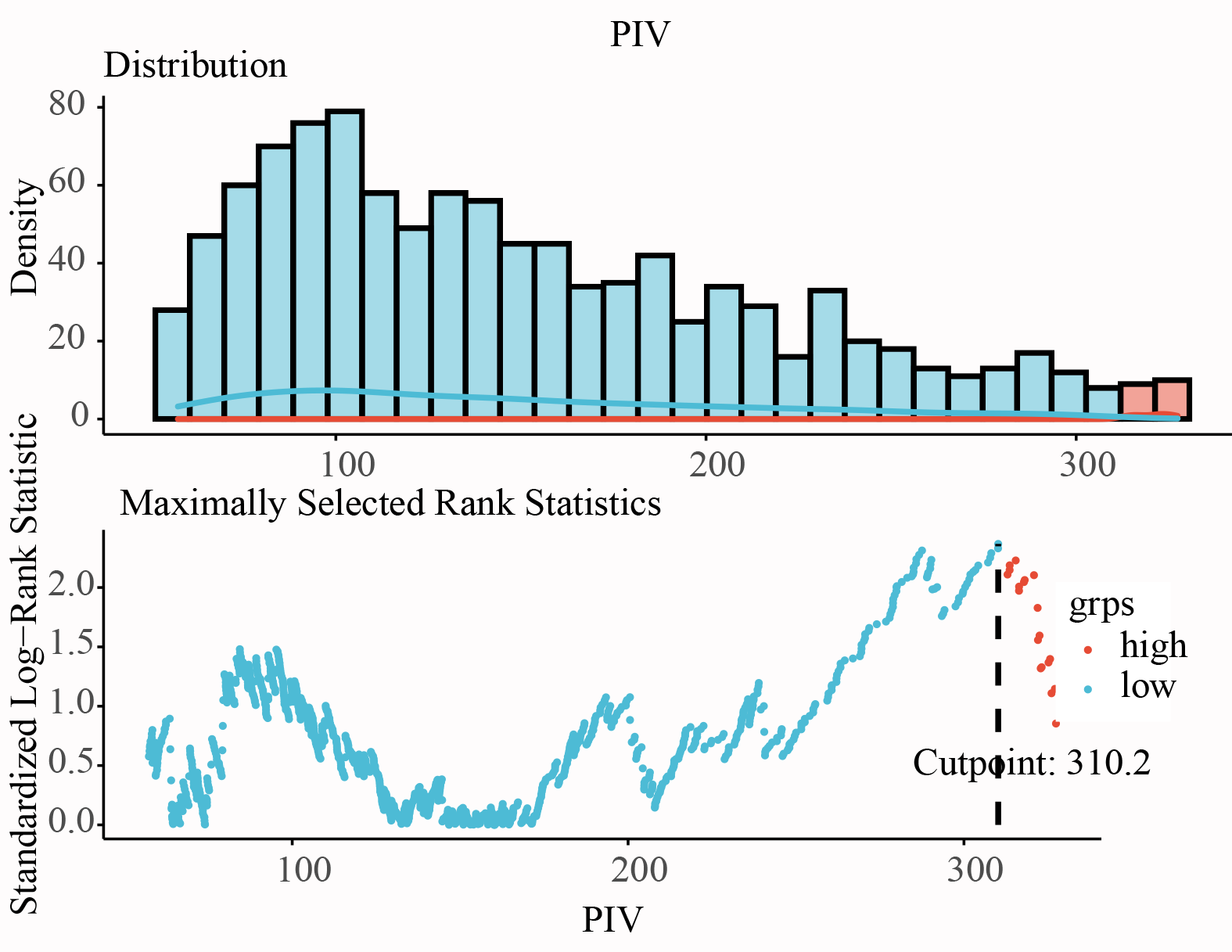

Supplement: Supplementary Figure 1 — The optimal cut-off of PIV for overall survival in the whole cohort by using maximally selected rank statistics. [file Image_1.tif]

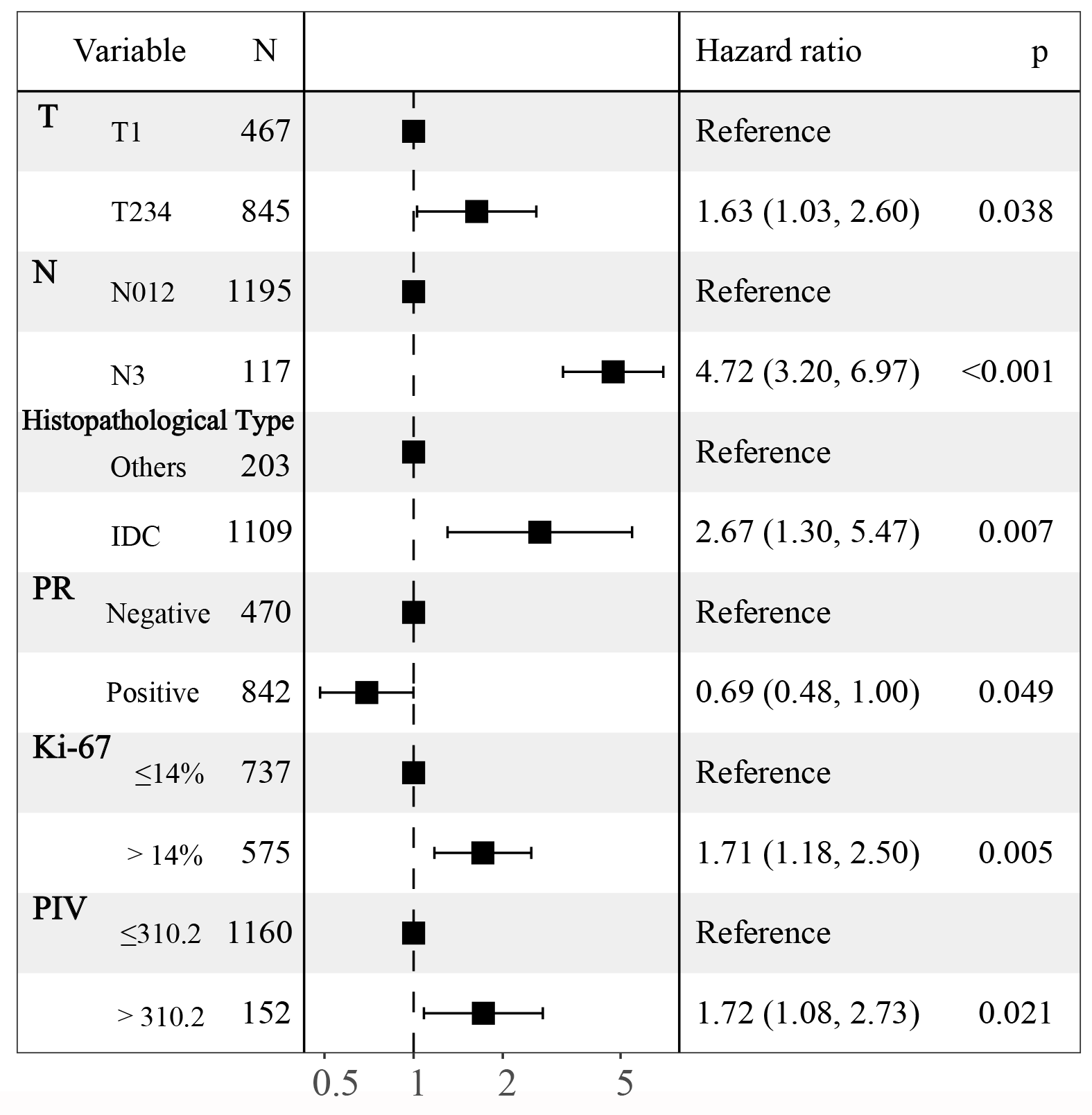

Supplement: Supplementary Figure 2 — The forest plot of the results of multivariable regression analysis. [file Image_2.tif]

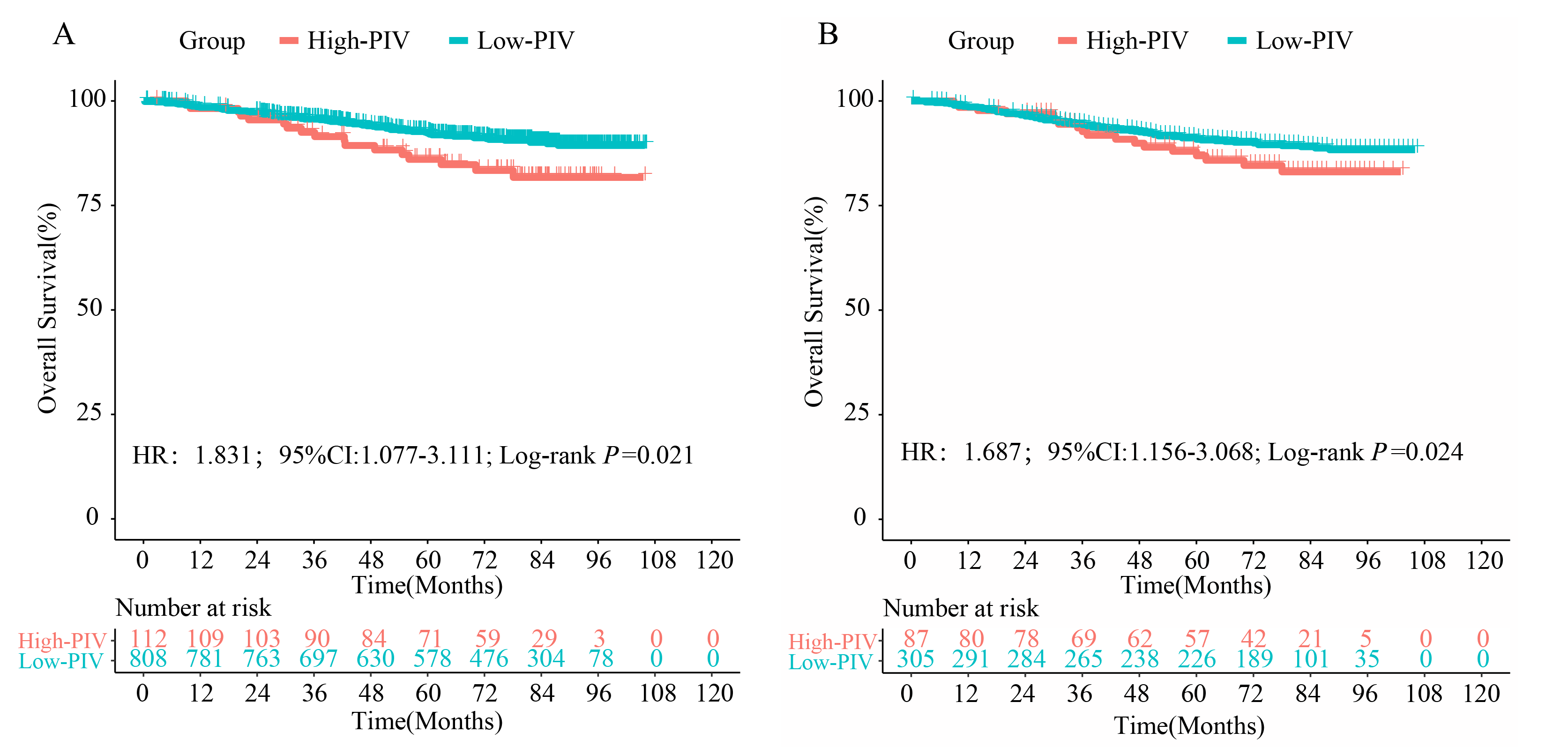

Supplement: Supplementary Figure 3 — Kaplan-Meier survival curves with breast cancer after surgery between the high-PIV group and low-PIV group (A) in the training set and (B) in the validation set. [file Image_3.tif]

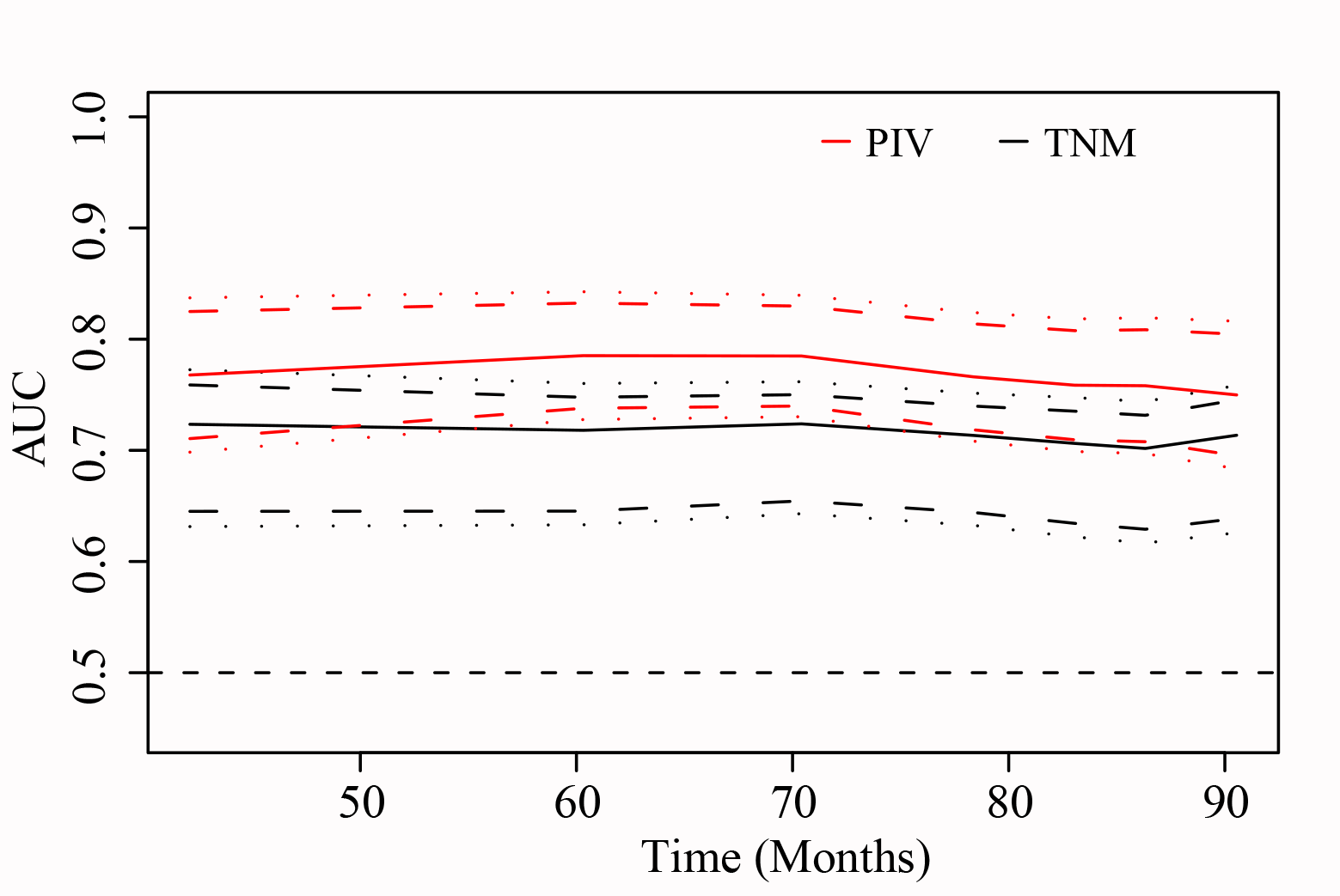

Supplement: Supplementary Figure 4 — Time-dependent ROC curve compared with PIV and the TNM staging system. [file Image_4.tif]

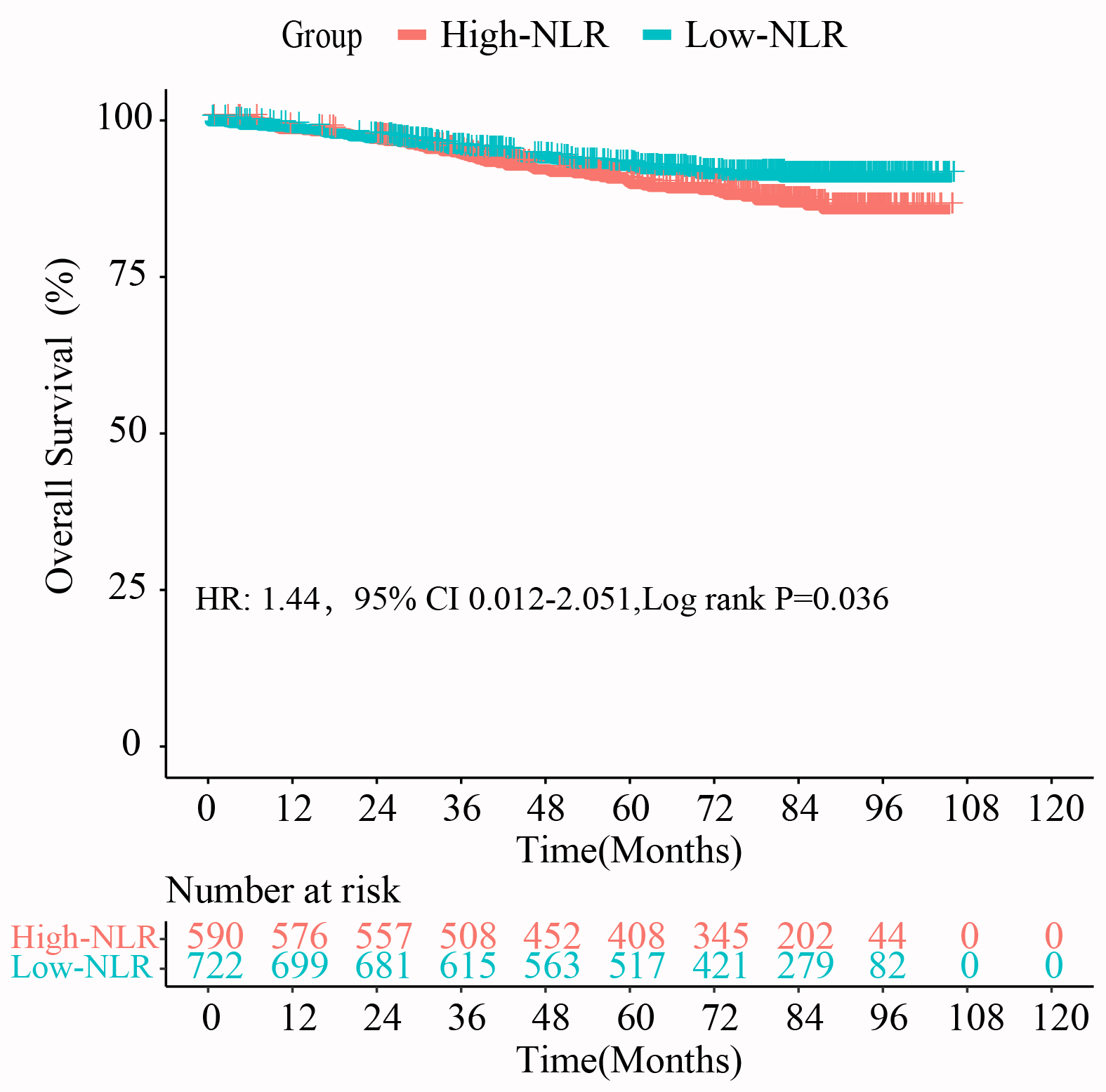

Supplement: Supplementary Figure 5 — Kaplan-Meier survival curves of breast cancer patients after surgery between the high-NLR group and low-NLR group in the whole cohort. [file Image_5.tif]

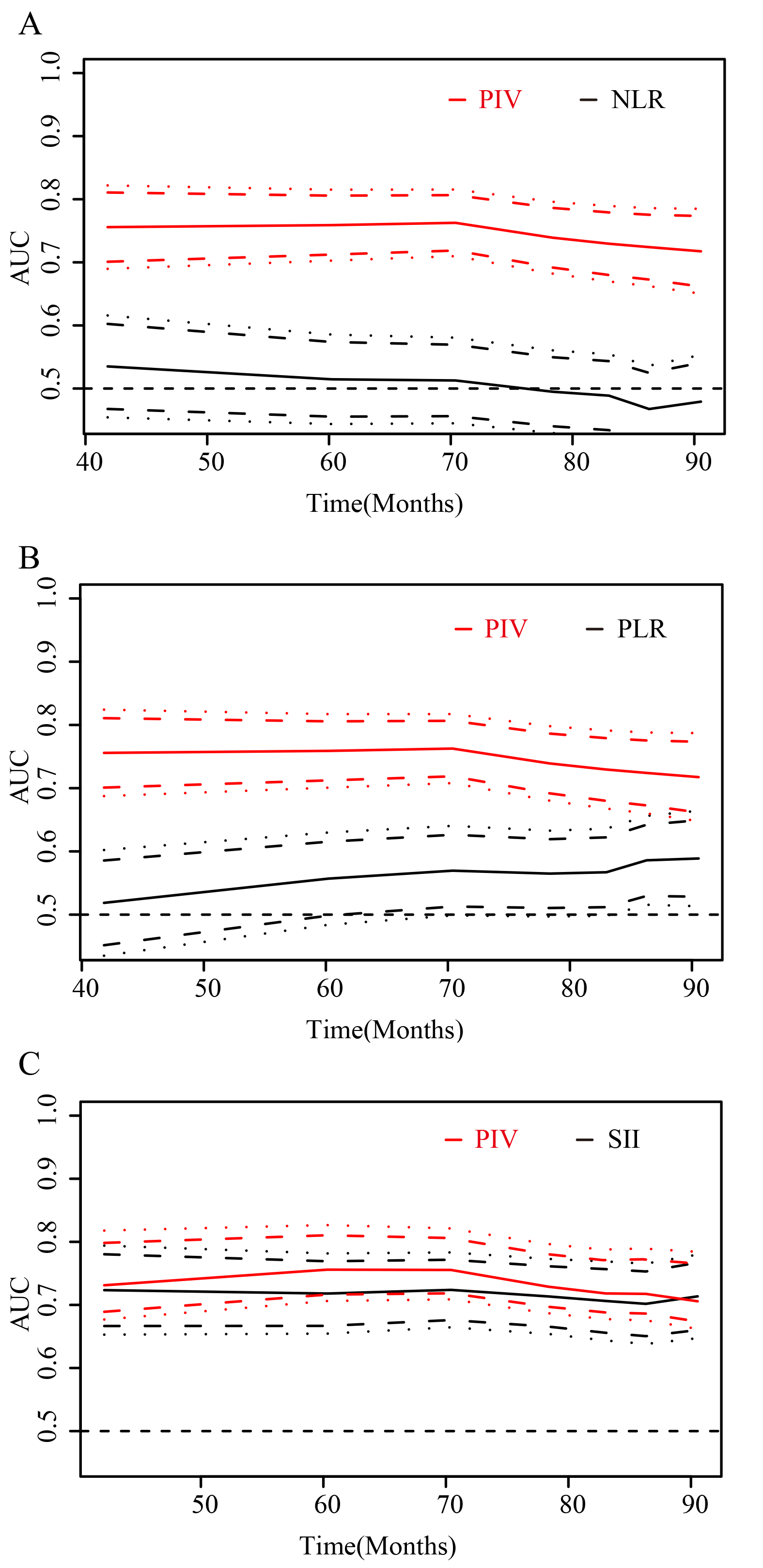

Supplement: Supplementary Figure 6 — Time-dependent ROC curve compared with PIV and (A) NLR, (B) PLR, (C) SII. [file Image_6.tif]
