# Supplementary material for: Pan-Immune-Inflammation Value: A New Prognostic Index in Operative Breast Cancer
Source: Front Oncol. 2022 Apr 13;12:830138. doi: 10.3389/fonc.2022.830138 (PMC9043599; doi:10.3389/fonc.2022.830138)
Supplement: Supplementary file 7 [file Table_1.docx]

**Table S1.** **Univariate and multivariate analysis of overall survival in the training set.**

| Characteristic | Univariate analysis  Hazard ratio(95%CI) | *P* | Multivariate analysis  Hazard ratio(95%CI) | *P* |
| --- | --- | --- | --- | --- |
| Age (years) | 1.546(0.678-1.792) | 0.521 | - | - |
| T stage | 2.013(1.391-2.875) | ＜0.001^*^ | 1.790(1.452-2.980) | 0.044^*^ |
| N stage | 7.326(4.109-9.385) | ＜0.001^*^ | 6.203(4.382-7.815) | <0.001^*^ |
| Histopathological Type | 1.807(1.526-3.778) | 0.003^*^ | 2.354(1.817-7.051) | 0.005^*^ |
| ER status | 0.695(0.434-0.787) | 0.001^*^ | 0.819(0.704-2.681) | 0.508 |
| PR status | 0.705(0.517-0.811) | 0.002^*^ | 0.850(0.583-1.215) | 0.056 |
| HER-2 status | 1.384(1.007-2.857) | 0.010^*^ | 1.476(0.913-1.578) | 0.189 |
| Ki-67 | 3.018(2.166-4.137) | ＜0.001^*^ | 3.601(2.750-4.068) | 0.008^*^ |
| NLR group | 1.893 (1.348-3.982) | 0.038^*^ | 2.193 (0.745-2.802) | 0.080 |
| PLR group | 1.692 (0.817-2.109) | 0.065 | - | - |
| SII group | 1.493 (0.779-1.990) | 0.089 | - | - |
| PIV group | 1.831(1.077-3.111) | 0.021^*^ | 1.963(1.367-3.212) | 0.039^*^ |

A Cox proportional hazards model was used to conduct multivariate analyses. All variables were transformed into categorical variables. HRs of variables were calculated as follows: Age (>48 y vs ≤48 y); T stage (T1 vs T234); N stage (N012 vs N3); Histological Type (Invasive ductal carcinoma vs others); ER (Negative vs Positive); PR (Negative vs Positive); HER-2 (Negative vs Positive); Ki-67 (≤14% vs >14%); NLR group (≤1.99 vs >1.99); PLR group (≤160.25 vs >160.25); SII group (≤642.23 vs >642.23); PIV group (≤310.20 vs >310.20).

***Abbreviations***: *P < 0.05; CI = confidence interval; ER, estrogen receptor; PR, progesterone receptor; HER2, human epidermal growth factor receptor-2; NLR, neutrophil-to-lymphocyte ratio; PLR, platelets-to-lymphocyte ratio; SII, systemic immune-inflammation index; PIV, pan-immune-inflammation value.

#According to the Eighth edition of the UICC/AJCC staging system.
