# Supplementary material for: Pan-Immune-Inflammation Value: A New Prognostic Index in Operative Breast Cancer
Source: Front Oncol. 2022 Apr 13;12:830138. doi: 10.3389/fonc.2022.830138 (PMC9043599; doi:10.3389/fonc.2022.830138)
Supplement: Supplementary file 8 [file Table_2.docx]

**Table S2.** **Univariate and multivariate analysis of overall survival in the validation set.**

| Characteristic | Univariate analysis  Hazard ratio(95%CI) | *P* | Multivariate analysis  Hazard ratio(95%CI) | *P* |
| --- | --- | --- | --- | --- |
| Age (years) | 1.698(0.401-2.013) | 0.760 | - | - |
| T stage | 1.705(1.254-2.181) | ＜0.001^*^ | 1.301(0.836-1.935) | 0.062 |
| N stage | 9.307(3.658-9.960) | ＜0.001^*^ | 7.272(5.387-9.018) | <0.001^*^ |
| Histopathological Type | 1.799(1.373-3.112) | ＜0.001^*^ | 1.655(1.349-4.141) | 0.008^*^ |
| ER status | 0.460(0.332-0.805) | 0.002^*^ | 0.698(0.593-3.107) | 0.091 |
| PR status | 0.819(0.699-0.905) | 0.003^*^ | 0.944(0.505-1.289) | 0.073 |
| HER-2 status | 2.010(1.572-3.430) | 0.029^*^ | 1.702(0.885-2.013) | 0.111 |
| Ki-67 | 2.090(1.356-4.117) | ＜0.001^*^ | 3.981(2.183-4.606) | 0.033^*^ |
| NLR group | 1.254 (1.031-2.675) | 0.041^*^ | 2.180 (0.638-4.382) | 0.076 |
| PLR group | 1.202 (0.561-1.884) | 0.072 | - | - |
| SII group | 1.809 (0.941-2.104) | 0.068 | - | - |
| PIV group | 1.687(1.156-3.068) | 0.024^*^ | 1.570(1.200-2.651) | 0.043^*^ |

A Cox proportional hazards model was used to conduct multivariate analyses. All variables were transformed into categorical variables. HRs of variables were calculated as follows: Age (>48 y vs ≤48 y); T stage (T1 vs T234); N stage (N012 vs N3); Histological Type (Invasive ductal carcinoma vs others); ER (Negative vs Positive); PR (Negative vs Positive); HER-2 (Negative vs Positive); Ki-67 (≤14% vs >14%); NLR group (≤1.99 vs >1.99); PLR group (≤160.25 vs >160.25); SII group (≤642.23 vs >642.23); PIV group (≤310.20 vs >310.20).

***Abbreviations***: *P < 0.05; CI = confidence interval; ER, estrogen receptor; PR, progesterone receptor; HER2, human epidermal growth factor receptor-2; NLR, neutrophil-to-lymphocyte ratio; PLR, platelets-to-lymphocyte ratio; SII, systemic immune-inflammation index; PIV, pan-immune-inflammation value.

#According to the Eighth edition of the UICC/AJCC staging system.
